# Supplementary material for: Performance of different nebulizers in clinical use for Pressurized Intraperitoneal Aerosol Chemotherapy (PIPAC)
Source: PLoS One. 2024 May 2;19(5):e0300241. doi: 10.1371/journal.pone.0300241 (PMC11065249; doi:10.1371/journal.pone.0300241)
Supplement: Attachment — Submitted filename: Comments to authors.pdf [file pone.0300241.s001.pdf]

Highlights of the manuscript:

The authors have tested clinically-operated nebulizing nozzles for PIPAC: HurriChem^{TM}; MCR-4 TOPOL®; QuattroJet) for i) technical design and principle of operation, ii) operational pressure as a function of the liquid flow rate, iii) droplet distribution via laser diffraction spectrometry, iv) spray cone angle, spray cone form as well as horizontal drug deposition by image-metric analyses and v) chemical resistance via exposing to a cytostatic solution and chemical composition by means of spark optical emission spectral analysis.

The authors have done a detailed and sound study of PIPAC nozzles which can make it easier to choose the best nozzle for chemotherapy in the future. It can help manufacturers and clinicians both in making better choices regarding cancer-type-specific PIPAC nozzle devices.

Accept after minor revisions

The authors have done a good job and the subject of the work in itself is exploration worthy and holds promising applications. PIPAC devices have become important for chemotherapy. This device has shown promise in improving median survival, especially in the case of ovarian and colorectal metastasis (https://www.ncbi.nlm.nih.gov/pmc/articles/PMC8683254).

A few suggestions to make the manuscript more meaningful are mentioned below:

1. What is the basis for choosing four commercial nebulizers from all the available options? What are the other available nozzles for PIPAC? Can we include a table of such nozzles and explain the choices made in the manuscript?
2. Lin 94: there is a question mark, please remove it. Also, write briefly about the "original nozzle technology in the same paragraph.
3. Line 72: Please give an introductory detail of the IDEAL-D framework somewhere in the manuscript to make it easy for the readers. A reference has already been given which is appreciated.
4. How is the mass median diameter generated from the nozzles related to the efficacy of treatment? Are we expecting a similar kind of droplet distribution when popular chemotherapeutic drugs like cisplatin, etc will be used? How will the aerosolization and droplet distribution change with changing molecular weight of the drugs? Can we think of some *in vivo* studies on cancer tissue slices or a plate-based assay to test the efficacy of cell survival? How are the functional parameters explored related to the killing the cancer cells, are there any related studies? Please include a little bit of such discussion in the manuscript to make it easy to be understood by people of the interdisciplinary fields.
