# Supplementary material for: Performance of different nebulizers in clinical use for Pressurized Intraperitoneal Aerosol Chemotherapy (PIPAC)
Source: PLoS One. 2024 May 2;19(5):e0300241. doi: 10.1371/journal.pone.0300241 (PMC11065249; doi:10.1371/journal.pone.0300241)
Supplement: Attachment — Submitted filename: PONE-D-23-07734_response_letter_reviewer_final.pdf [file pone.0300241.s002.pdf]

Cover Letter – Submission ID: - PONE-D-23-07734

Revision of our submission “Performance of different nebulizers in clinical use for Pressurized Intraperitoneal Aerosol Chemotherapy (PIPAC)”

Dear Mr. Mohammad Mehdi Rashid,

We would like to thank the editorial team for handling our manuscript and the reviewers for their comments.

Below, please find our point-by-point responses to the reviewers’ comments, and attached, the revised version of our manuscript with tracked changes.

We hope that you find our responses and revisions satisfactory. In case of further questions or concerns, please do not hesitate to contact us.

Sincerely,

Professor Dr. med. Urs Giger-Pabst, Dipl.-Ing. Daniel Göhler and co-authors

Responses to reviewer comments

Reviewer comments are printed in *italics*.

Reviewer 1

Reviewer #1: 1. Overview

This study aimed to compare the technical performance of four commercial nebulizer nozzles used for Pressurized Intraperitoneal Aerosol Chemotherapy (PIPAC). The analysis included technical design, operational pressure, droplet size distribution, spray cone angle and form, horizontal drug deposition, chemical resistance, and chemical composition. The Nebulizer showed a similar technical design and performance to the original PIPAC nozzles, while the other nozzles demonstrated deviation from the original nozzles. The study recommends that PIPAC nozzles that deviate from the current standard undergo bioequivalence testing and follow the IDEAL-D framework before clinical use.

Major Comments

1.1 *The paper presents a meaningful and rigorous study of the four types of nebulizer nozzles. The experiment results and suggestions are important to guide new pre-clinical screening to determine its equivalency. In addition to the experiment and discussion in the paper, it would be helpful to include the information below.*

Response: We would like to thank the reviewer for this encouraging comment!

Action taken: none

1.2 *The paper mentions multiple times that the aerosol generation principle is different between A/B, C, and D. It would be helpful to elaborate further on how the aerosol is generated in each device. A zoom-in schematic diagram of the liquid aerosolization in each nebulizer may further assist in the understanding of their performance difference.*

Response: Thank you for your remark, but please note that we do not like to show highly-resulted details on the nozzle design to avoid non-controlled replication of them by third parties. In our opinion the technical construction and the associated mode of operation is

described in sufficient detail on the basis of the given construction drawings (page 7, lines 174 to 193,).

Action taken: none

***1.3** The nebulizers are eventually used for PIPAC. It would be important to cite and summarize the drug delivery requirement and guidelines of the PIPAC. The discussion would be more meaningful in the context of its actual application environment.*

Response: This is of course a valid remark. There are already some information concerning some parameters in the submitted manuscript (e.g.: 3rd paragraph of chapter 3.2, 6th paragraph in the discussion). But please note the following. At the time of this study, the four PIPAC nozzles tested here were commercially available and clinically used for PIPAC and PITAC treatments. In the meantime, additional models have become available on the market. The nozzles were tested according to the instructions for use of the respective nozzle manufacturers. Our current work is a purely technical descriptive and comparative ex vivo study.

We would like to point out that PIPAC is carried out in the context of an off-label use. There is no scientific evidence regarding optimized nozzle specifications, aerosol characteristics, capnoperitoneal pressure, duration of capnoperitoneal pressure, medication, medication dosage, etc. A superiority of PIPAC therapy in oncological outcome compared to intraperitoneal liquid chemotherapy or systemic chemotherapy has not been clinically proven so far. Therefore, we consider it of little value to discuss expert opinions within the scope of our manuscript.

Action taken: none

***1.4** In addition to suggesting equivalency studies, the result here could further guide the development of new nebulizer structures that combine the advantage and avoid the drawback of each design. At the end of the discussion, the author could provide such suggestions for new device development.*

Response: In principle, the authors agree with that remark of the reviewer. Of course, there are some papers available addressing the improvement of PIPAC, but for the current approval of the method this can also have an opposite effect. Since also a further reviewer asked for the same information, the authors decided to prepare a new paragraph.

Action taken: The following paragraph was added on page 14, line 379ff:

"The extent to which the PIPAC nozzle designs and thus its' spray characteristics affect the spatial distribution, penetration depth and tissue concentration of drugs has hardly been investigated so far. Göhler et al. [22] compared the performance of the original PIPAC nozzle with that of a two-substance nozzle in a post-mortem pig model and found that due to the even more finer droplet size distribution of the latter one, the spatial drug distribution of ^{99m}Tc pertechnetate as well as the penetration depth of doxorubicin becomes significantly more homogeneously. Moon et al. [19] presented a nozzle (DreamPen®) with a spray cone angle of 77° that rotates 30° around its vertical axis during operation within the abdominal cavity. The direct comparison with the original PIPAC nozzle in an in-vivo pig model showed an improved spatial drug distribution pattern of methylene blue and significantly higher penetration depths or tissue concentrations of doxorubicin in the peritoneum [20]. In a recent study, Braet et al. [21] administered nanoparticles in an ex-vivo model via an original PIPAC nozzle and a multidirectional nebulizer (Medspray®), each with and without additional electrostatic aerosol precipitation. It was found that the addition of electrostatic aerosol precipitation improves the spatial distribution of the nanoparticles and their penetration depth into the tissue, regardless of the nozzle used. Additionally, the operation of the multidirectional nozzle showed that the spatial distribution pattern of the nanoparticles was more homogeneous than for the conventional PIPAC nozzle. To our knowledge, neither the two-substance nozzle nor the DreamPen® or the Medspray® nebulizer is approved for clinical use. In summary, the mentioned preclinical studies show that there is large space for advanced technologies that can improve the efficacy of PIPAC. But the impact on oncologic outcome is still unclear and requires extensive preclinical and clinical research in the near future."

1.5 *Page 5 line 120, throughout the testing, the horizontal nozzles were mostly sealed and not tested. It would be helpful to include data where horizontal nozzles are opened while the axis nozzle is sealed. Combining the two experiments could provide a whole picture of the nozzle D performance.*

Response: The authors agree with the reviewer's remark, but the geometric limitations of the operated laser diffraction spectrometer did not allow a characterization of the aerosols from the lateral orifices. Of course, we tried to analyze the aerosol formed by the lateral orifices (with a higher distance to the laser beam), but the optics of the instrument became

rapidly contaminated so that no valid analyses could be performed. Thus, only analyses of the aerosol from the axial orifice perpendicular to the laser beam was possible. However, since the four orifices of the QuattroJet are exactly identical in construction, it is assumed that the granulometric aerosol characteristics at the lateral orifices is identical.

Action taken: To emphasize this analytical limitation a second time in the manuscript, the following sentence was added the end of chapter 2.3: "Due to geometric limitations of the operated laser diffraction spectrometer, only the aerosol that leaves the axial orifice could be analyzed for nozzle D, while the lateral orifices had to be sealed."
To underline that the orifices and twist bodies are identical in design, the following action was taken: page 9, line 4f was changed from "with four twist bodies in "with for identical orifices and twist bodies"

1.6 *Fig 4, it is apparent from the bottom panel that the density of aerosols has a special distribution. Further image analysis of the color intensity and integration of the intensity over the whole area could give a quantitative estimation of the drug dose. If possible, such integration over 3D space is even better.*

Response: We agree with the reviewer that 3D deposition analyses would provide more details about the spatial drug distribution pattern and we will try to perform such experiments in future analyses. Note that nozzles used for this study were cut open (as stated in chapter 2.1) after the experiments for analyzing their design and thus are not available for further analyses. In this study, 2D deposition analyses were only performed as side experiments to visualize the spray cone configuration of each nozzle. 2D deposition analyses showed that there was no relevant drug deposition in the center of the spray cone at nozzle C due to the hollow spray cone. Furthermore, previous analyses on the base of gravimetric analyses and laser diffraction spectrometry have shown that for the conventional PIPAC nozzle A, most (97.5% of the volume) of the nebulized drug directly impacts on the peritoneum below the nozzle orifice (see Ref. 6, 8). This observation largely holds true for nozzles B and C as well.

Action taken: None

1.7 *Page 6 line 158, it is alarming that nozzle C forms rust over long exposure to the cytostatic solution. However, given their different standard operation conditions (see Page 6 line 138), nozzle*

C has the highest flow rate. To make a fair comparison assuming the same drug dose, nozzle C's testing time or salt concentration might be scaled down compared to nozzle A/B/D.

Response: All nozzles were exposed to a standardized chemotherapy solution for 12 hours. The development of iron oxide was then observed at nozzle C after 12 days. All other nozzles showed no signs of iron oxide formation after this time. The formation of iron oxide in nozzle C prompted us to perform an independent metallurgical analysis, which showed that the cause of iron oxide formation in nozzle C was the use of steel that did not meet surgical steel standards due to its high sulfur content. The different flow rate of the nozzles does not seem to be relevant. Nozzle D is also operated at 1.5 ml/s. However, this nozzle showed no iron oxide formation in the test described above. In the discussion, it is described that the 12-hour exposure of the nebulizer D to the chemotherapy solution does not correspond to everyday clinical practice and that the observation of iron oxide formation alone is not sufficient to draw conclusions about a possible hazard potential for the patient in everyday clinical practice.

Action taken: None

Reviewer #2:

This study aimed to compare four commercial nebulizer nozzles used for Pressurized Intraperitoneal Aerosol Chemotherapy (PIPAC). The Nebulizer showed similar performance to the original PIPAC nozzles, while the HurriChemTM offered a finer aerosol, MCR-4 TOPOL® had larger droplets, and QuattroJet improved spatial drug distribution. The authors recommend conducting bioequivalence testing and adhering to the IDEAL-D framework before implementing new PIPAC nozzles with different performances in routine clinical use.

2.1 *Introduction: The introduction provides a good background on PIPAC, its clinical significance, and the need for standardized nozzles. However, it would be beneficial to include a clearer statement of the research objective or hypothesis to guide the readers better.*

Response: The authors agree with the reviewers' comment. A more detailed statement about the study aim is now given in the Introduction.

Action taken: The following statement is now included on page 4, line 96 to 101: "Based on the methodological findings on the technical characterization of the original PIPAC nozzle [6], this study aims to comparatively characterize the technical design, granulometric performance and spray characteristics (spray angle/cone) of all until the beginning of 2023 existing/known clinically operated nozzles for PIPAC. In addition, the chemo-resistance and metallurgical properties of the different nozzles were investigated by an independent, government-accredited testing agency.

2.2 *The study design and methodology seem appropriate for the research objective. However, additional details on the experimental setup, such as the type of high-pressure injector used and any potential variations in environmental conditions (temperature, humidity), should be included to ensure reproducibility by other researchers.*

Response: Note that we already mentioned all relevant information on the operated instruments in chapter "2 Material and Methods". For example, on page 5, line 113/114 you will find the full specification of the operated high-pressure injector.
We agree with the reviewer that the ambient conditions can have an effect especially on aerosol formation.

Action taken: At the beginning of chapter 2, the following sentence was added: "Excepting the investigation concerning chemical resistance and composition, all analyses took place in a non-technical-ventilated room (volume = 120.0 m3) at a temperature of ϑ = (20.3 ± 1.3) °C, a relative humidity of φ = (38.2 ± 3.7) % and a pressure of p = (101.6 ± 3.3) kPa".

2.3 *While the results are presented in a clear manner, it would be beneficial to include statistical analyses to determine the significance of the differences observed between the nozzles, especially when comparing operational pressure and droplet size distribution.*

Response: The authors agree with the reviewers' remark. For the sake of readability, we omitted that in the submitted manuscript. Due to the request of the reviewer, we decided to discuss at least the particle size data of the analysed nozzles when operated at manufacturer-recommended conditions.

Action taken: The following three sentences were added at the end of the 4th paragraph in chapter 3.2: "A statistical examination of the determined mass median diameters at manufacturer-recommended operation conditions (circled data in Figure 3 b) shows on the basis of a Kruskal-Wallis H test [35] that the determined data for the analysed nozzles are significantly different ($p_{A/B/C/D}$ = 0.000104). Paired comparisons of the different nozzles using the Mann-Whitney test [36, 37] showed in the case of nozzle B (HurriChemTM) and D (QuattroJet) quasi no significant difference ($p_{B/D}$ = 0.26). In all other cases, determined mass median diameters of the different nozzles were found to be significant different to each other ($p_{A/B}$ = 0.0101, $p_{A/C}$ = 0.0002, $p_{A/D}$ =0.0101, $p_{B/C}$ = 0.0038, $p_{C/D}$ = 0.0038). In addition, the reference list was expanded by 3 further references.

2.4 *The chemical resistance of the nozzles is an important factor, especially when considering exposure to cytostatic solutions. A more detailed discussion of the implications of iron oxide formation in nozzle C and its potential impact on nozzle performance or patient safety should be included.*

Response: We agree with the reviewer. The formation of iron oxide in nebulizer D due to the use of non-surgical steel is of concern. However, we note that iron oxide formation was observed after an exposure period of 12 hours in chemotherapy solution with an additional duration of 12 days. This does not correspond to the clinical use of nebulizer C. We take this into account by discussing on page 12 that the testing does not correspond to clinical

use, but some risk to patients from iron oxide cannot be ruled out at this time. We further mention that manufacturers and also regulatory authorities have to take into account such circumstances whether the materials used comply with the requirements.
In order to investigate a possible hazard from the use of nozzle C, further extensive toxicological testing is required. However, these is not part of this current study. In the near future, however, we will be able to present such data in a separate paper on this subject. In the meantime, we do not wish to prejudge or speculate on this issue of iron oxide formation.
Thus, we believe that our current statement on this is correct and defensible at this time.

Action taken: None

2.5 *Clinical Relevance: While the technical and operational comparisons are well-done, it would be valuable to discuss the potential clinical implications of the observed differences in nozzle performance. How might these differences affect drug delivery and patient outcomes during PIPAC procedures?*

Response: We would like to point out that there are currently no reliable data showing that PIPAC could lead to a better clinical outcome compared to liquid intraperitoneal chemotherapy or systemic chemotherapy. Furthermore, there is no preclinical or clinical data for nozzles B, C and D that could allow a technical, let alone preclinical or clinical evaluation at this point in time. It can be assumed for nozzle B, since it is actually a replica of nozzle A, that similar pharmacological and clinical results can be achieved as for nozzle A. For nozzle C there are also no preclinical data or clinical data that determine the drug distribution, drug penetration depth or tissue concentrations. We even state on page 11, line 313 to 316, that the hollow spray cone design of nozzle C does not per se improve drug distribution, penetration and in-tissue concentrations.
Furthermore, there are no technical, preclinical or clinical data specifications from the manufacturer for nozzle B. A technical comparison between the different nozzles was so far not possible as there is a lack of data. Therefore, our present study is a purely comparative technical study to get an insight about the performance of the different nebulizers. However, these technical data are not sufficient to draw final conclusions about their pharmacological performance or even their clinical outcome. This is the main aim of the current study. To explore any impact on the clinical outcome by the use of different

PIPAC nozzles we promote that the nozzles used for PIPAC will be specified in the international PIPAC database.

Action taken: Nevertheless, as suggested by the reviewer, we now have included the following additional statements in the discussion. On page on page 14 the following statement is now given: “The extent to which the PIPAC nozzle designs and thus its’ spray characteristics affect the spatial distribution, penetration depth and tissue concentration of drugs has hardly been investigated so far. Göhler et al. [22] compared the performance of the original PIPAC nozzle with that of a two-substance nozzle in a post-mortem pig model and found that due to the even more finer droplet size distribution of the latter one, the spatial drug distribution of 99mTc pertechnetate as well as the penetration depth of doxorubicin becomes significantly more homogeneously. Moon et al. [19] presented a nozzle (DreamPen®) with a spray cone angle of 77° that rotates 30° around its vertical axis during operation within the abdominal cavity. The direct comparison with the original PIPAC nozzle in an in-vivo pig model showed an improved spatial drug distribution pattern of methylene blue and significantly higher penetration depths or tissue concentrations of doxorubicin in the peritoneum [20]. In a recent study, Braet et al. [21] administered nanoparticles in an ex-vivo model via an original PIPAC nozzle and a multidirectional nebulizer (Medspray®), each with and without additional electrostatic aerosol precipitation. It was found that the addition of electrostatic aerosol precipitation improves the spatial distribution of the nanoparticles and their penetration depth into the tissue, regardless of the nozzle used. Additionally, the operation of the multidirectional nozzle showed that the spatial distribution pattern of the nanoparticles was more homogeneous than for the conventional PIPAC nozzle. To our knowledge, neither the two-substance nozzle nor the DreamPen® or the Medspray® nebulizer is approved for clinical use. In summary, the mentioned preclinical studies show that there is large space for advanced technologies that can improve the efficacy of PIPAC. But the impact on oncologic outcome is still unclear and requires extensive preclinical and clinical research in the near future.”

***2.6** Limitations: Include a section on limitations to acknowledge any potential biases or shortcomings in the study. For instance, limitations related to the sample size, choice of test liquids, or specific measurement techniques should be discussed.*

Response: The authors agree with the proposal of the reviewer that information on study limitations enhance the content of the paper.

Action taken: A further paragraph that contain remarks and limitation to this study are added at the end of the chapter 4:

"It is pointed out that the following remarks and limitations have to be taken into account when evaluating the results of this study. First, one has to keep in mind that all barometric and granulometric analyses were performed on the basis of an aqueous solution of glucose (5 wt.-%). These data lose their validity, if liquids with deviating viscosity (such as drug carrier gels, nanoparticles, antibody solutions, etc.,) are nebulized for research or clinical purposes. Second, only one specimen of each type of nozzle was examined at least three time, i.e., the impact of manufacturing tolerances on the performance of the nozzles is unknown. Third, due to geometric limitations of the operated laser diffraction spectrometer, only the aerosol that leaves the axial orifice of nozzle D (QuattroJet) could be analysed. Fourth, the operated laser diffraction spectrometer (PW180-C spray particle size analyser, Jinan K-Ring Technology Co., Ltd, Shandong, China) was successfully validated against a second laser diffraction spectrometer (HELOS/KR-H2487, Sympatec GmbH, Clausthal-Zellerfeld, Germany) on the basis of one standard PIPAC nozzle. Ultimately, when a nebulizing system for PIPAC is clinically used, the responsibility for compliance with national/supranational regulations for the use of medical devices lies with the user."

2.7 *Since the angioinjector is not yet standardized, it is necessary to investigate the potential changes that may occur when using different types of devices. Therefore, a comprehensive evaluation of the effects of using alternative devices is required. This includes assessing any potential variations in operational parameters, droplet size distributions, spray cone angles, drug deposition areas, and chemical resistance compared to the angioinjector. Understanding these differences will be crucial for determining the clinical implications and safety of using different devices for Pressurized Intraperitoneal Aerosol Chemotherapy (PIPAC).*

Response: The present study did not explore the impact of different high-pressure injectors on the technical performance of different nozzles used for PIPAC. However, we would like to point out that the nozzles used for PIPAC in the last 12 years did not have CE/FDA certification for PIPAC therapy but only for the nebulization of aqueous fluids in body cavities in the context of minimally invasive surgery (laparoscopy /thoracoscopy). The nozzles were therefore used off-label for PIPAC. Also, the high-pressure injectors for PIPAC were used off-label. Although there is now a CE certified high-pressure injector for PIPAC therapy available, it should be mentioned that all CE certified high-pressure

injectors allow a maximum tolerance deviation of +/- 0.3% of the respective value/parameter. This is checked annually during mandatory inspection/maintenance in countries of the European Community. This means that all approved and regularly evaluated high-pressure injectors are suitable for PIPAC in off-label use. Given the small permitted deviations, there are no relevant changes with regard to granulometric characteristics, spray angle, etc.

Action taken: None

2.8 *To establish a direct clinical relevance and connection, it is essential to consider parameters such as penetration depth and tissue concentration. However, it is evident that these factors may present limitations in this study. Since the angioinjector has not been standardized, variations in operational parameters and droplet characteristics among different devices could potentially impact penetration depth and drug tissue concentration. Thus, to fully understand the clinical implications and efficacy of using alternative devices for Pressurized Intraperitoneal Aerosol Chemotherapy (PIPAC), further research and validation studies are warranted. These investigations should focus on the actual clinical outcomes, including tumor response rates, patient outcomes, and adverse events, while considering the impact of different device designs and performance characteristics on drug delivery to the targeted tissues.*

Response: We would like to point out again that this study only represents a comparative technical characterization. We agree with the reviewer that the spatial distribution pattern, penetration depth and tissue concentrations need to be examined in a further step in order to establish a possible connection with the nozzle design, the aerosol generated and the resulting pharmacology and clinical outcome. A first such comparative in-vivo study in a pig model on the local and systemic pharmacology of the different nozzles was recently carried out by our research group. The results of this study will be published and will hopefully provide further insights about the individual nozzle systems.

However, we do not agree with the Reviewer that the use of different high-pressure injectors will have an impact on the clinical outcome of PIPAC. We consider the influence of the high-pressure injector on the performance of the nozzles and thus on a different clinical outcome to be unlikely. As already stated above under point 2.7, CE-approved devices that are evaluated annually in accordance with the regulations may only show deviations of +/- 0.3%. An influence on the performance of the nozzles or an influence on the clinical outcome can be classified as low when using different high-pressure injectors.

Such minimal deviations are unlikely to be detectable in a clinical study, especially with regard to the oncological outcome. As already mentioned, we recommend that the PIPAC nozzle type used should be recorded in the International PIPAC database in order to be able to record a possible influence on the clinical outcome.

Action taken: None

2.9 *Addressing the regulation issues related to nozzles in different countries would indeed add more depth to the content. Currently, there might be varying regulatory frameworks and standards governing the approval and use of nebulizer nozzles for PIPAC in different countries. Understanding these variations is crucial as it may impact the availability, accessibility, and usage of different nozzle types globally. Additionally, these regulatory differences may also affect the acceptance and adoption of newer nozzle technologies in specific regions, making it necessary to navigate through regulatory hurdles before implementing them in routine clinical practice. Therefore, exploring the regulatory landscape in different countries will provide valuable insights into the challenges and opportunities surrounding the usage of various PIPAC nozzles worldwide.*

Response: We agree with the reviewer that different countries also have different regulatory mechanisms for the approval of medical devices. The manufacturers of nebulizer A, C and D are member states of the European Union. These products therefore primarily have CE class IIa approval. The manufacturer of nebulizer B is based in the USA. This product has FDA and CE class IIa approval. According to our current knowledge, FDA approval will also be available for nebulizer A and D shortly. We do not have any information regarding this for nebulizer C.

The discussion of the individual regulations in the different countries and their possible influences on product development and clinical applicability of the different nebulizers is not the subject of this work. Furthermore, such an in-depth discussion would go beyond the scope of this work.

It is therefore the responsibility of the clinical user to ensure that the nebulizer to be used complies with the legal requirements of the relevant country for clinical use.

Action taken: To take the reviewers remark into account, we added the following sentence at the end of the last paragraph in chapter 4:

“Ultimately, when a nebulizing system for PIPAC is clinically used, the responsibility for compliance with national/supranational regulations for the use of medical devices lies with the user.”

Reviewer#3

The authors have tested clinically-operated nebulizing nozzles for PIPAC: HurriChemTM; MCR-4 TOPOL®; QuattroJet) for i) technical design and principle of operation, ii) operational pressure as a function of the liquid flow rate, iii) droplet distribution via laser diffraction spectrometry, iv) spray cone angle, spray cone form as well as horizontal drug deposition by image-metric analyses and v) chemical resistance via exposing to a cytostatic solution and chemical composition by means of spark optical emission spectral analysis.

The authors have done a detailed and sound study of PIPAC nozzles which can make it easier to choose the best nozzle for chemotherapy in the future. It can help manufacturers and clinicians both in making better choices regarding cancer-type-specific PIPAC nozzle devices.

Accept after minor revisions

The authors have done a good job and the subject of the work in itself is exploration worthy and holds promising applications. PIPAC devices have become important for chemotherapy. This device has shown promise in improving median survival, especially in the case of ovarian and colorectal metastasis (https://www.ncbi.nlm.nih.gov/pmc/articles/PMC8683254).

A few suggestions to make the manuscript more meaningful are mentioned below:

***3.1** What is the basis for choosing four commercial nebulizers from all the available options? What are the other available nozzles for PIPAC? Can we include a table of such nozzles and explain the choices made in the manuscript?*

Response: At the time of this study, the four PIPAC nozzles characterized here were in regular clinical use. To our very best knowledge we are not aware about other nozzle systems that were regularly used for PIPAC.

Recently, an additional nozzle has become available that has CE approval for PIPAC application. Due to its recent appearance, this nozzle could no longer be included in this study. Details about the tested nozzles in this present study are already summarized in Table 1.

Action taken: To underline that all existing/known clinically operated nozzles up to the beginning of 2023 were characterized, the 3rd paragraph of the introduction was change from: "On the basis of the methodological findings regarding the technical characterization of the original PIPAC nozzle [8], the current study deals with the comparative performance characterization of four commercial nebulizers as nowadays used for PIPAC" into "Based

on our previous methodological findings on the technical characterization of the original PIPAC nozzle [6], this follow-up study aims to comparatively characterize the technical design, granulometric performance data and spray characteristics (spray angle/cone) of all until the beginning of 2023 existing/known clinically operated nozzle systems for PIPAC treatment. In addition, the chemo-resistance and metallurgical properties of the different nozzles were investigated by an independent, government-accredited testing agency.".

***3.2** Line 94: there is a question mark, please remove it. Also, write briefly about the "original nozzle technology in the same paragraph.*

Response: The reviewer is right.

Action taken: The question mark has been removed. The following details about the original PIPAC nozzle technology is now given on page 4: "The original nozzle which was used for off-label PIPAC is a CE certified class IIa device to aerosolize aqueous drug solutions. The nebulizer produces a polydisperse, bimodal aerosol with a volume-weighted median droplet diameter of 25 µm with a full spray cone of approximately 70°. Ex-vivo gravimetric studies demonstrated an inhomogeneous spatial distribution pattern with more than 86.0 vol.-% of the administered chemotherapy deposited by inertial impaction directly below the nozzle outlet on a circular surface with a diameter of 15 cm. Calculations reveal that under the given flow conditions the droplet size for a homogenous intra-abdominal drug distribution during PIPAC therapy should be approximately 1.2 µm. However, 97.5 vol.-% of the aerosolized liquid is composed of droplets with a diameter of > 3 µm [6]."

***3.3** Line 72: Please give an introductory detail of the IDEAL-D framework somewhere in the manuscript to make it easy for the readers. A reference has already been given which is appreciated.*

Response: The authors agree with the reviewers' comment that additional information regarding the IDEAL-D framework can improve the understanding.

Action taken: On page 12, the following statement is now given: IDEAL-D is an independent network of clinicians and scientists that aims to provide a framework for evaluating innovations in surgical techniques from conception, through technical development, research, evaluation and long-term follow-up. The central tenet of this

framework is that development and evaluation can and should be done together in an orderly and logical manner that balances innovation and safety. The evaluation for interventional therapies in the IDEAL-D framework includes four stages comprising the preclinical phase (stage 0), first-in-human studies (stage 1), prospective developmental/exploratory studies (stage 2), large randomized controlled trials/or equivalent studies (stage 3) and long-term surveillance studies and registries (stage 4).

3.4 *How is the mass median diameter generated from the nozzles related to the efficacy of treatment? Are we expecting a similar kind of droplet distribution when popular chemotherapeutic drugs like cisplatin, etc will be used? How will the aerosolization and droplet distribution change with changing molecular weight of the drugs? Can we think of some in vivo studies on cancer tissue slices or a plate-based assay to test the efficacy of cell survival? How are the functional parameters explored related to the killing the cancer cells, are there any related studies? Please include a little bit of such discussion in the manuscript to make it easy to be understood by people of the interdisciplinary fields.*

Response: There are no data indicating a correlation between droplet size and cytotoxicity. However, the size of the aerosol droplets is crucial for the quality of the spatial distribution and thus also for the cytotoxic effect in the abdominal cavity [see reference 6 and 22]. All PIPAC nozzles are class CE IIa and approved for off-label use nebulization of aqueous solutions in laparoscopic or thoracoscopic surgery. Since the cytostatic drugs currently used for PIPAC, such as doxorubicin, cisplatin and doxorubicin, are nebulized in aqueous solution, our investigations regarding granulometry and spray cone are valid. At this point it must be mentioned that laser diffraction spectrometry with toxic substances can only be carried out with considerable effort. For safety reasons, nozzle characterisation is therefore carried out with non-toxic substances such as glucose 5%. Glucose 5% is used in PIPAC and is therefore an ideal test substance. As soon as the viscosity of the liquids to be atomized differ, our data are no longer valid. In this case, the substance to be nebulized must be tested separately for its granulometric properties or for its pharmacological activity (in the case of biologics/nanoparticles).

With regard to the testing of PIPAC, different nozzle types and their influence on cytotoxicity, we provide guidance on page 14 [ref 22-31] on ex- and in vivo small and large animal models used to assess drug distribution, penetration depth, cytotoxicity, and cytotoxicity.

Action taken: The following further explanations were included in the manuscript on page 14:

"The extent to which the PIPAC nozzle designs and thus its' spray characteristics affect the spatial distribution, penetration depth and tissue concentration of drugs has hardly been investigated so far. Göhler et al. [22] compared the performance of the original PIPAC nozzle with that of a two-substance nozzle in a post-mortem pig model and found that due to the even more finer droplet size distribution of the latter one, the spatial drug distribution of 99mTc pertechnetate as well as the penetration depth of doxorubicin becomes significantly more homogeneously. Moon et al. [19] presented a nozzle (DreamPen®) with a spray cone angle of 77° that rotates 30° around its vertical axis during operation within the abdominal cavity. The direct comparison with the original PIPAC nozzle in an in-vivo pig model showed an improved spatial drug distribution pattern of methylene blue and significantly higher penetration depths or tissue concentrations of doxorubicin in the peritoneum [20]. In a recent study, Braet et al. [21] administered nanoparticles in an ex-vivo model via an original PIPAC nozzle and a multidirectional nebulizer (Medspray®), each with and without additional electrostatic aerosol precipitation. It was found that the addition of electrostatic aerosol precipitation improves the spatial distribution of the nanoparticles and their penetration depth into the tissue, regardless of the nozzle used. Additionally, the operation of the multidirectional nozzle showed that the spatial distribution pattern of the nanoparticles was more homogeneous than for the conventional PIPAC nozzle. To our knowledge, neither the two-substance nozzle nor the DreamPen® or the Medspray® nebulizer is approved for clinical use. In summary, the mentioned preclinical studies show that there is large space for advanced technologies that can improve the efficacy of PIPAC. But the impact on oncologic outcome is still unclear and requires extensive preclinical and clinical research in the near future."

In the new last paragraph of chapter 4, we comment now also the data validity: "First, one has to keep in mind that all barometric and granulometric analyses were performed on the basis of an aqueous solution of glucose (5 wt.-%). These data lose their validity, if liquids with deviating viscosity (such as drug carrier gels, nanoparticles, antibody solutions, etc.,) are nebulized for research or clinical purposes."
